# Significance of p53, p27, Ki-67, E-cadherin, and HER2 expression in upper urinary tract urothelial carcinoma

**DOI:** 10.1186/s43046-020-00047-z

**Published:** 2020-09-15

**Authors:** Nabiha Missaoui, Ahlem Bdioui, Atika Baccouche, Oussema Belkacem, Wissem Hmida, Moncef Mokni, Sihem Hmissa

**Affiliations:** 1 Faculty of Sciences and Techniques of Sidi Bouzid, Kairouan University, Kairouan, Tunisia; 2 Pathology Department, Farhet Hached University Hospital, 4000 Sousse, Tunisia; 3 Pathology Department, Sahloul University Hospital, 4002 Sousse, Tunisia; 4 Urology Department, Sahloul University Hospital, 4002 Sousse, Tunisia

**Keywords:** Upper urinary tract urothelial carcinoma, p53 expression, Positive surgical margin, Survival

## Abstract

**Background:**

The study investigated the expression and the clinicopathological significance of p53, p27, Ki-67, E-cadherin, and HER2 in upper urinary tract urothelial carcinomas (UTUC) from Tunisian patients. We performed a retrospective study of 66 UTUC. Main clinicopathological features were reported. The expression of p53, p27, Ki-67, E-cadherin, and HER2 was investigated by immunohistochemistry on whole tissue section.

**Results:**

Expression of p53, Ki-67, p27, E-cadherin, and HERE2 was reported in 36.4%, 69.7%, 90.9%, 100%, and 0% of cases, respectively. p53 expression was associated with stage (*p* = 0.001), positive surgical margin (*p* = 0.005), and shorter recurrence-free survival (RFS; Log Rank test, *p* = 0.026). Ki-67 and p27 expression was associated with stage (*p* < 0.001 and *p* = 0.001, respectively) and grade (*p* < 0.001 and *p* = 0.001, respectively). Using Kaplan-Meier test, the positive surgical margin was associated with shorter RFS compared to free surgical margin (Log Rank test, *p* = 0.031). Moreover, in univariate Cox regression analysis, surgical margin (*p* = 0.041; HR 0.325, 95% CI 0.110–0.956) and p53 expression (*p* = 0.035; HR 0.328, 95% CI 0.116–0.925) were the significant factors associated with RFS.

**Conclusions:**

Together, our findings suggest that positive surgical margin and p53 expression were potential prognostic factors of UTUC since both were associated with shorter RFS in Tunisian patients.

## Background

Upper urinary tract urothelial carcinomas (UTUC) are relatively rare malignancies, accounting for approximately five to 10% of all urothelial cancers [1]. The clinicopathological characteristics and the prognosis of UTUC are completely different from those of bladder cancer. In fact, UTUC are often aggressive with poor prognosis compared to bladder cancer [1]. Due to their anatomical particularities, including a thin muscle layer, proximity to the kidney, and rich lymphatic drainage, tumor invasion affects significantly tumor progression and distant metastasis [1]. Nevertheless, standard pathological factors, including histopathological stage and grade and lymph node metastasis, were actually the only prediction factors of the prognosis [1]. Because of their low frequency and clinical presentation heterogeneity, limited surveys have been accomplished on UTUC; as a result, the molecular mechanisms of tumor invasion, recurrence, and prognosis remain ambiguous [1–5]. In addition, different behaviors are still reported even among patients with the same stage and/or grade [1, 2]. New specific and reliable biomarkers could improve the prognostic accuracy, outcome prediction, or treatment and would benefit patients [2].

To accurately improve the oncological outcomes of patients, previous reports analyzed several prognostication factors and identified novel clinicopathological parameters, including patient age, multifocality, concomitant in situ carcinoma, tumor architecture and size, and variant histology [1, 2]. In addition, the prognostic interest of factors involved in cell cycle and apoptotic pathways, cell proliferation and adhesion, metastasis, and microsatellite instability has been investigated [2].

In our study, we explored the clinicopathological value of p53, p27, Ki-67, E-cadherin, and HER2 in UTUC. p53 protein is implicated in genomic stability, cell cycle, and apoptosis. *p53* alterations were described in several malignancies and correlated with mediocre prognosis in various cancers [3]. In UTUC, p53 might be a helpful marker for tumoral recurrence and survival prediction [3–5].

p27 belongs to the Cip/Kip family and inhibits cyclin-dependent kinases. Normal level of p27 expression is involved in controlling cell proliferation and tumor progression. Decreased or absent p27 expression has been described as a potent negative prognostic indicator in several tumors, including bladder cancer [6]. The p27 role in UTUC has been investigated with contrasting results [7, 8].

The classic marker of cellular proliferation, Ki-67, is a main prognostic indicator of tumor recurrence, aggressiveness, and progression. In bladder cancer, Ki-67 was linked to tumor stage, grade, and recurrence [9]. By contrast, in UTUC, the Ki-67 significance remains controversial [8, 10, 11].

E-cadherin is a transmembrane glycoprotein implicated in tissue integrity. Loss of E-cadherin expression is involved in tumor de-differentiation and invasiveness caused by the dissociation of cells from tissue structures [12]. The signification of E-cadherin in UTUC has been investigated by some contrasting studies [8, 13, 14].

Unlike bladder carcinomas, only few reports analyzed the overexpression and/or gene amplification of the human epidermal growth factor receptor type 2 (HER2) in UTUC and its clinical significance is still contentious [15, 16].

To further explore these rare tumors, we examined the expression and the clinicopathological significance of p53, p27, Ki-67, E-cadherin, and HER2 in UTUC from Tunisian patients.

## Methods

### Tissue samples

We carried out a retrospective study of 66 UTUC diagnosed in the Pathology Department of our University Hospital, during 2000–2015. Samples with unavailable clinicopathological data were excluded from the present study. This study was approved by the local Human Ethics Committee of our University Hospital, and it conformed to the provisions of the Declaration of Helsinki.

Two pathologists reviewed hematoxylin and eosin-stained sections of all cases, using the latest edition of the TNM/AJCC classification. Based on the 2016 World Health Organization (WHO) classification [17], cases were histopathologically divided into low-grade and high-grade tumors. Tissues of all selected cases had been routinely fixed in 10% buffered formalin and paraffin embedded.

### Clinicopathological data

Clinicopathological data was collected using clinical records of patient. Age at diagnosis, gender, cancer discovery circumstances, tumor location and focality, histopathological stage and grade, surgical margin status, nodal and vascular invasion, treatment, tumoral recurrence, and patient outcome were recorded.

### Immunohistochemistry

The immunoexpression of p53, p27, Ki-67, E-cadherin, and HER2 was carried out by immunohistochemistry on whole sections as we recently described [17]. In brief, after antigen unmasking at 95 °C for 40 min, the endogenous peroxidase activity was blocked using 3% hydrogen peroxide. Primary antibodies were next added at room temperature (30 min) as described in Table 1. The staining was revealed by the Envision+ Dual Link System HRP kit (Dako, code K4063). Appropriate positive controls for each immunostaining reaction were performed according to manufacturer’s directives.

**Table 1:** Immunohistochemistry conditions and evaluation

Expression	Provenance	Clone	Dilution	Retrieval solution	Positive immunostaining
p53	DAKO	Do-7	1:50	Citrate 0.01 M pH 6	Nuclear staining in ≥ 50% of tumor cells
p27	DAKO	Sx5368	1:25	Citrate 0,01 M pH 9	Nuclear staining in ≥ 10% of tumor cells
Ki-67	DAKO	Mib1	1:50	Citrate 0,01 M pH 6	Nuclear staining in ≥ 10% of tumor cells
E-cadherin	DAKO	NCH-38	1:30	Citrate 0,01 M pH 6	Membranous staining in ≥ 10% of tumor cells
HER2	DAKO	Polyclonal (A0485)	1:300	Citrate 0,01 M pH 6	Membranous staining in ≥ 30% of tumor cells

Immunohistochemistry evaluation was independently performed by two pathologists. Only staining of tumor cells was observed. The immunostaining was assessed semi-quantitatively by considering the percentage of positive cells and staining intensity as described previously [8, 18]. Ki-67 expression was classified as low (≤ 20% of cell nuclei stained positive for Ki-67), high (> 20%), and negative (0%) [10].

### Statistical analysis

All statistical analyses were conducted with SPSS version 19.0 (IBM Corp., Armonk, NY, USA). Fisher’s exact probability test was used to analyze the link between the protein expression and the clinicopathological features. Recurrence-free survival (RFS) curves were generated using the Kaplan-Meier estimates, with the Log Rank test being applied for the comparison of survival curves. Furthermore, hazard ratios (HR) and 95% confidence intervals (CI) computed from univariate and multivariable Cox regression models were used to investigate the relationship between studied features. Probability values (*p*) of .05 or less were considered statistically significant.

## Results

### Clinicopathological findings

The patient’ age at diagnosis ranged between 39 and 90 years with a median age of 67 years. There was a male predominance (sex ratio 2.7:1). Among patients, 25.7% were smokers. Diagnosis time ranged from 0 (incidental finding) to 120 months with an average of 12 months. Tumors were localized in the renal pelvis or calyces (75.8%) and in the ureter (24.2%). Twenty-nine patients had a history of chronic pyelonephritis and eleven patients had an antecedent bladder tumor at the time of diagnosis.

All patients were diagnosed with papillary urothelial carcinoma. There were five cases (7.6%) in stage pTa (papillary, noninvasive tumors), 14 cases (21.2%) in stage pT1 (tumors invading the submucosa), 19 cases (28.8%) in stage pT2 (tumors invading the muscularis), 23 cases (34.8%) in stage pT3 (tumors invading beyond the muscularis or renal parenchyma), and five cases (7.6%) in stage pT4 (tumors metastasizing the regional lymph node or a distant site). According to histopathological grade, tumors were classified as low grade (36.4%) and high grade (63.6%). Low-grade tumors were budding, polypoid or sessile, papillary, brown, and crumbly. High-grade tumors were ulcerative, infiltrative, white-gray with yellowish areas, necrotic, and hemorrhagic. Tumors were unifocal in 48.5% of cases and multifocal in the remaining cases (51.5%). Vascular invasion was detected in 19.7% of UTUC cases. Among the 66 patients, lymph node dissection was performed in 22 (33.3%) patients, and 6 of them had pathologically node-positive disease (27.3%).

The surgical management was applied for 63 patients, including simple nephrectomy (23 patients), total nephroureterectomy (22 patients), nephroureterectomy with collar excision (5 patients), endoscopic resection (2 patients), and ureterectomy (2 patients). Three patients received adjacent chemotherapy in addition to surgery. Two patients received chemotherapy and only one patient received radiotherapy as palliative treatment.

### Immunohistochemistry results

**Fig. 1:**
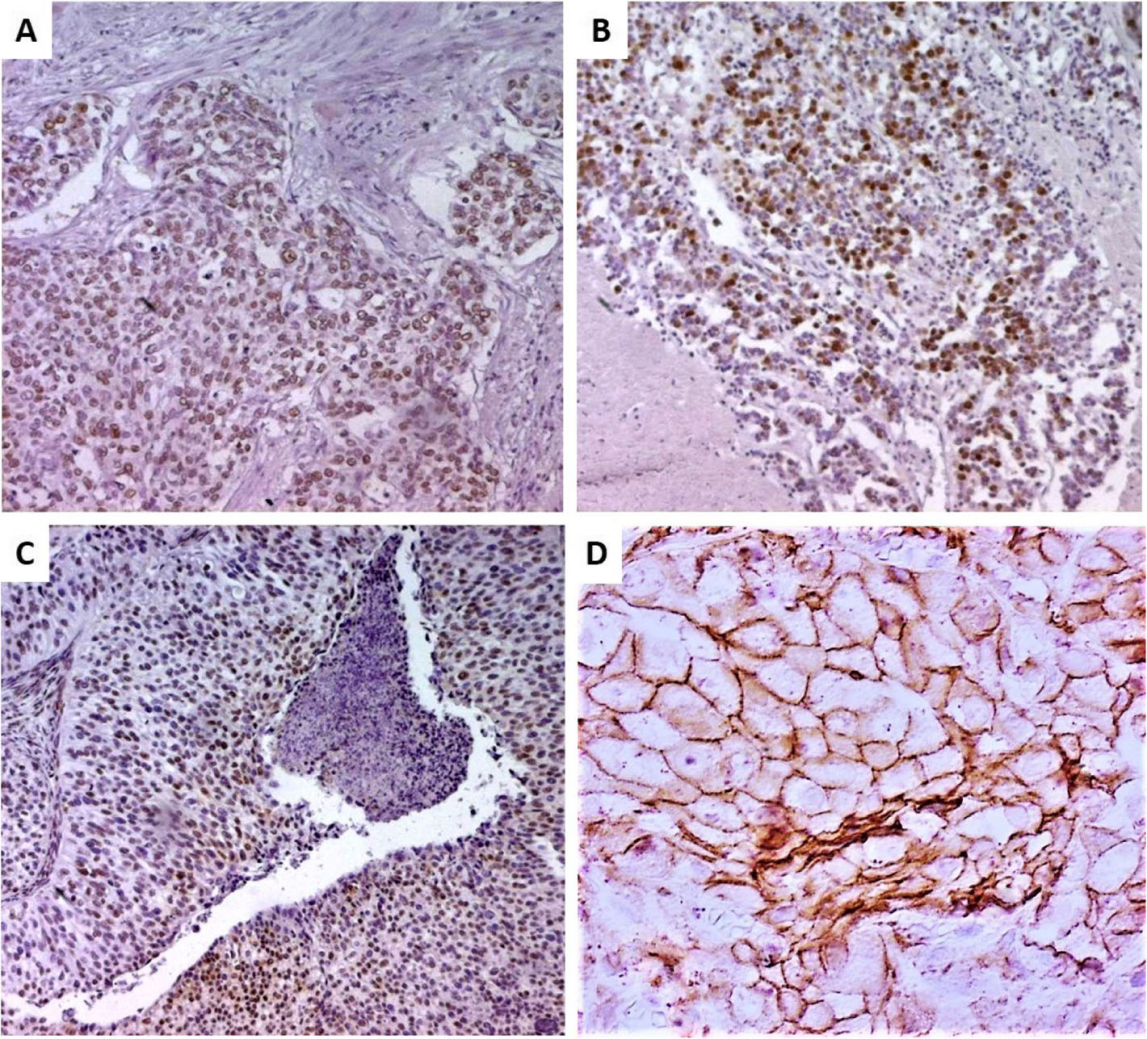
Immunohistochemical findings of UTUC. **a** Nuclear expression of p53 (× 100). **b** Nuclear expression of p27 (× 100). **c** Nuclear expression of Ki-67 (× 100). **d** Membrane expression of E-cadherin (× 400)

The expression of p53, p27, Ki-67, E-cadherin, and HER2 was summarized in Table 2. p53 expression was observed in only 36.4% of cases (Fig. 1a). Nuclear expression of p27 protein was recognized in the majority of tumors (90.9%, Fig. 1b). Ki-67 expression was reported in 69.7% of cancers (Fig. 1c). Based on the percentage of cell nuclei stained positive for Ki-67, Ki-67 expression was low in 24 UTUC and high in the remaining tissue samples (*n* = 22). All studied tumor cases exhibited E-cadherin expression (Fig. 1d). However, no HER2 positivity was detected in all UTUC.

**Table 2:** Clinicopathological features and expression of p53, p27, Ki-67, E-cadherin, and HER2 in UTUC

Parameters	Category	p53		p27		Ki-67		E-cadherin	HER2
		Negative *n* (%)	Positive*n* (%)	Negative*n* (%)	Positive *n* (%)	Negative *n* (%)	Positive *n* (%)	Positive *n* (%)	Negative *n* (%)
All cases	*n* = 66	42 (63.6)	24 (36.4)	6 (9.1)	60 (90.9)	20 (30.3)	46 (69.7)	66 (100)	66 (100)
Gender	Male (*n* = 49)	34 (51.5)	15 (22.7)	3 (4.5)	46 (69.7)	15 (22.7)	34 (51.5)	49 (74.2)	49 (74.2)
	Female (*n* = 17)	8 (12.1)	9 (13.6)	3 (4.5)	14 (21.2)	5 (7.6)	12 (18.2)	17 (25.8)	17 (25.8)
Age (years)	≤ 60 (*n* = 14)	9 (13.6)	5 (7.6)	2 (3)	12 (18.2)	5 (7.6)	9 (13.6)	13 (19.7)	13 (19.7)
	61–70 (*n* = 27)	20 (30.3)	7 (10.6)	3 (4.5)	24 (36.4)	11 (18.2)	16 (24.2)	28 (42.4)	28 (42.4)
	≥ 71 (*n* = 25)	13 (19.7)	12 (18.2)	1 (1.5)	24 (36.4)	4 (6.1)	21 (31.8)	25 (37.9)	25 (37.9)
Stage	≤ pT1 (*n* = 19)	17 (25.7)	2 (3)	6 (9.1)	13 (19.7)	15 (22.7)	4 (6.1)	19 (28.8)	19 (28.8)
	pT2-pT4 (*n* = 47)	25 (37.9)	22 (33.3)^a^	0	47 (71.2)^a^	5 (7.6)	42 (63.6)^c^	47 (71.2)	47 (71.2)
Grade	Low (*n* = 24)	18 (27.3)	6 (9.1)	6 (9.1)	18 (27.3)	16 (24.2)	8 (12.1)	24 (36.4)	24 (36.4)
	High (*n* = 42)	24 (36.4)	18 (27.3)	0	42 (63.6)^a^	3 (4.5)	39 (59.1)^c^	42 (63.6)	42 (63.6)
Surgical margin	Healthy (*n* = 57)	40 (60.6)	17 (25.7)	4 (6.1)	53 (80.3)	16 (24.2)	40 (60.6)	57 (86.4)	57 (86.4)
	Positive (*n* = 9)	2 (3)	7 (10.6)^b^	2 (3)	7 (10.6)	3 (4.5)	6 (9.1)	9 (13.6)	9 (13.6)
Tumor focality	Unifocal (*n* = 34)	19 (28.8)	15 (22.7)	5 (7.6)	29 (43.9)	9 (13.6)	25 (37.9)	34 (51.5)	34 (51.5)
	Multifocal (*n* = 32)	23 (34.8)	9 (13.6)	1 (1.5)	31 (47)	11 (16.7)	21 (31.8)	32 (48.5)	32 (48.5)
Vascular invasion	Yes (*n* = 14)	7 (10.6)	7 (10.6)	0	14 (21.2)	0	14 (21.2)	14 (21.2)	14 (21.2)
	No (*n* = 52)	35 (53)	17 (25.7)	6 (9.1)	46 (69.7)	20 (30.3)	32 (48.5)^d^	52 (78.8)	52 (78.8)
Nodal invasion	N+ (*n* = 5)	1 (1.5)	4 (6)	0	5 (7.6)	0	5 (7.6)	5 (7.6)	5 (7.6)
	N_0_ (*n* = 18)	12 (18.2)	6 (9.1)	3 (4.5)	15 (22.7)	8 (12.1)	10 (15.1)	18 (27.3)	18 (27.3)
	Nx (*n* = 43)	29 (43.9)	14 (21.2)	3 (4.5)	40 (60.6)	12 (18.2)	31 (47)	43 (65.1)	43 (65.1)
Tumor recurrence	Yes (*n* = 15)	8 (12.1)	7 (10.6)	2 (3)	13 (19.7)	7 (10.6)	8 (12.1)	15 (22.7)	15 (22.7)
	No (*n* = 51)	34 (51.5)	17 (25.7)	4 (6.1)	47 (71.2)	13 (19.7)	38 (57.6)	51 (77.3)	51 (77.3)

Only significant results were indicated with a: *p =* 0.001, b: *p* = 0.005, c: *p* < 0.001, d: *p* = 0.007

Statistical analysis showed a significant association of positive p53 expression to tumor stage (*p* = 0.001), positive surgical margin (*p* = 0.005), and Ki-67 expression (*p* = 0.001). Positive p27 expression was associated with tumor stage and grade (*p* = 0.001 for both). In addition, positive Ki-67 expression (low and high) was significantly associated with tumor stage (*p* < 0.001) and grade (*p* < 0.001), vascular invasion (*p* = 0.006), and p27 expression (*p* = 0.032).

### Clinical outcome

Tumor progression was marked by a recurrence on the urinary tract for 13 cases (19.7%). Only one patient died of his cancer, which was initially classified as stage IV. Only one patient was lost to follow-up.

**Fig. 2:**
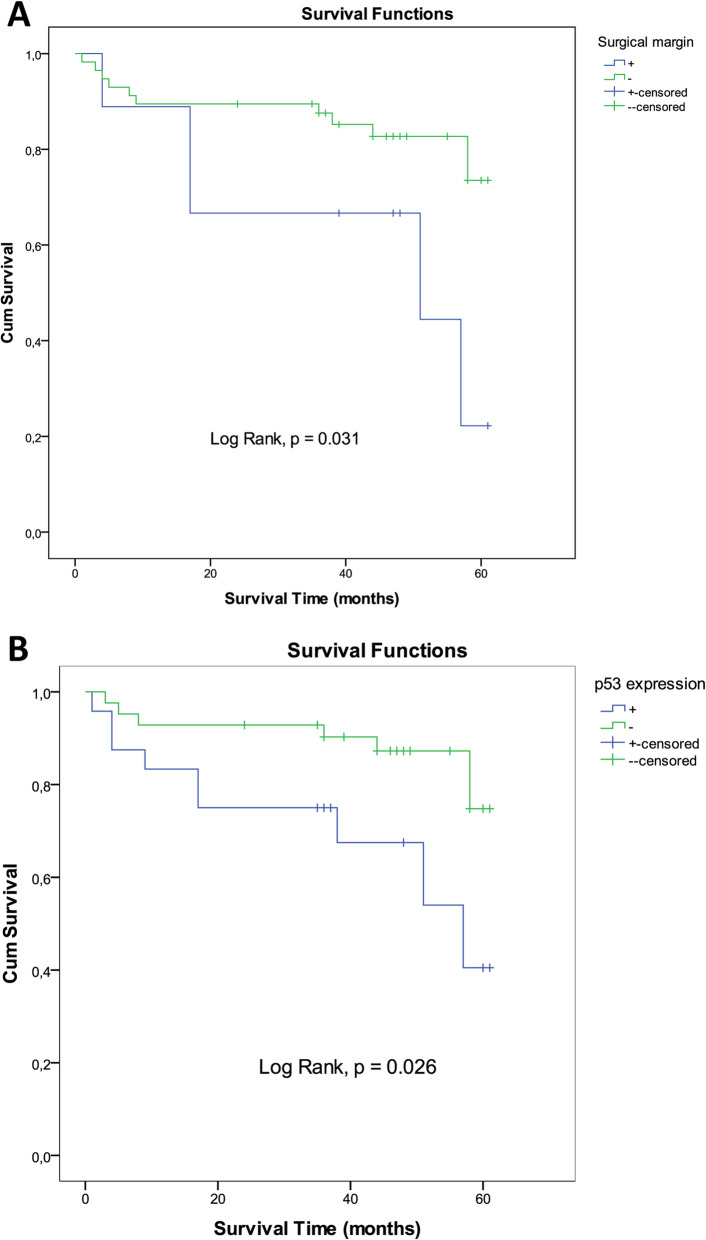
Kaplan-Meier survival curves for patients with UTUC according to surgical margin (**a**) and p53 expression (**b**). Using the Kaplan-Meier, surgical margin and p53 expression were significantly associated with RFS (*p* = 0.031 and *p =* 0.026, respectively; Log Rank test)

Using Kaplan-Meier test, positive surgical margin was associated significantly with shorter RFS (*p* = 0.031, Fig. 2a). However, no other significant findings were identified with patient age (*p* = 0.184), gender (*p* = 0.253), tumor location (*p* = 0.268) and focality (*p* = 0.944), tumor grade (*p* = 0.506) and stage (*p* = 0.762), vascular invasion (*p* = 0.962), and nodal invasion (*p* = 0.409).

Among the five studied proteins, the Kaplan-Meyer test showed that only positive p53 expression was associated significantly with shorter RFS (*p* = 0.026, Fig. 2b). No further significant findings were observed for the expression of Ki-67 (*p* = 0.466) and p27 (*p* = 0.669).

Table 3 presented the univariate and multivariable Cox regression analysis of RFS for patients with UTUC. The univariate analysis showed that surgical margin (*p* = 0.041; HR 0.325, 95% CI 0.110–0.956) and p53 expression (*p* = 0.035; HR 0.328, 95% CI 0.116–0.925) were the significant factors associated with RFS. Nonetheless, in multivariate analysis, no significant association was identified (Table 3).

**Table 3:** Univariate and multivariable analysis of recurrence-free survival for patients with UTUC

	Univariate			Multivariate		
	***p*** values	Hazard ratio	95% confidence interval	***p*** values	Hazard ratio	95% confidence interval
Patient age	0.078	1.913	(0.929–3.939)			
Gender	0.268	0.430	(0.097–1.911)			
Tumor stage	0.869	1.038	(0.664–1.624)			
Tumor grade	0.509	0.710	(0.257–1.960)			
Tumor focality	0.945	1.037	(0.374–2.875)			
Localization anatomic	0.282	2.264	(0.510–10.045)			
Surgery margin	0.041*	0.325	(0.110–0.956)	0.271	0.508	(0.152–1.700)
Nodal invasion	0.422	2.109	(0.341–13.061)			
Vascular invasion	0.962	1.031	(0.291–3.660)			
p53 expression	0.035*	0.328	(0.116–0.925)	0.142	0.420	(0.132–1.337)
Ki-67 expression	0.469	1.466	(0.520–4.130)			
p27 expression	0.671	1.382	(0.311–6.137)			

*Significant *p* values

## Discussion

The tumor stage and grade are the most recognized prognostic factors in UTUC [1, 2, 19–22]. However, herein, no significant association was identified with patients RFS. In the study of Olgac et al. [22], the stage was the most potent predictor of patient survival similar to the urothelial tumors of lower urinary tract. In a multi-institutional dataset of patients investigated by Novara et al. [19], the stage as well as the lymph nodes and the presence of synchronous muscle-invasive bladder cancer were independent predicting factors of cancer-specific survival. In addition, Langner et al. [20] found that stage was the only independent prognostic factor regarding disease-free survival. Two years later, in multivariate analysis of 190 consecutive invasive UTUC, these researchers considered that pT classification and vascular invasion were independent prognostic factors of metastasis-free survival [21].

In the present study, the positive surgical margin was associated significantly with shorter RFS, supporting its putative prognostic role in UTUC. Interestingly, Formont et al. [8] reported tumor recurrence and poor prognosis in all patients with positive surgical margins. Using univariate analysis, Olgac et al. [22] showed that margin status was significantly associated with patient survival. These results altogether highlight the importance of high quality of the initial tumor resection and checking margin status.

The prognostic significance of the cell cycle regulator, p53, in UTUC has been investigated previously with conflicting results [3–5, 8, 23]. In an early study, Terrell et al. [23] neglected any prognostic significance of p53 since there were no significant association with grade, stage, and cancer-specific survival. Ten years later, Fromont et al. [8] ignored as well the association between p53 and patient survival. Nonetheless, herein, only p53 expression was associated significantly with shorter RFS as well as with stage, positive surgical margin, and Ki-67 expression. Interestingly, previous studies reported significant correlations between p53 expression and advanced stage, high grade, and female gender as well as with disease-free, cancer-specific, and overall survival rates [3, 5]. Furthermore, p53 may be a useful marker to predict recurrence patterns and could be a prognosis predictor of UTUC in addition to tumor grade and growth pattern [4].

Currently, positive p27 expression was associated significantly with tumor stage and grade. However, we did not find any significant association with RFS. Similarly, in the early study of Nakanishi et al. [24], although p27 expression decreased significantly with stage and grade, no correlation was reported with overall and disease-free survival rates. Using tissue microarray technology, Fromont et al. [8] and Munari et al. [25] found also no significant prognostic interest of p27 expression in UTUC. However, more recently, although no association was described between p27 expression and tumor stage or grade, loss of p27 expression was correlated with tumor architecture and patient overall survival [7]. In fact, as described in papillary bladder carcinomas, Sarsik et al. [7] found loss of p27 expression in 33.3% of UTUC samples with invasive pattern while all noninvasive cases exhibited p27 expression. Similar to p27 expression loss, tumors with lower p27 expression displayed invasive growth pattern [7].

Ki-67 expression has been reported previously in 53 to 88% of UTUC [8, 10, 11, 26, 27]. In our study, we observed positive Ki-67 expression in 69.7% of cases. The significant association between the positive Ki-67 expression and the tumor stage, grade, and vascular invasion suggest the involvement of Ki-67 in UTUC invasiveness. Previously, a higher Ki-67 expression was found as well in T2-T4 tumor compared with Tis-T1 tumors and was associated with poor survival and high risk of disease progression [10]. Furthermore, a significant association was identified between Ki-67 overexpression and adverse clinicopathological features and poor prognosis in patients after radical nephroureterectomy [26], supporting its value as a promising indicator predicting survival [11]. In retrospective and prospective analyses, Krabbe et al. [27] considered that Ki-67 is an independent predictor of RFS in patients with high-grade UTUC. Nonetheless, herein, we found no significant association of Ki-67 expression with RFS.

Unlike previous reports, no significant prognostic value for E-cadherin expression was identified herein [8, 13, 14, 28]. In the Nakanishi et al. [28] study, E-cadherin expression was associated with stage and grade, pattern of growth, disease-free, and overall survival rates only in univariate analysis. Using tissue microarray technology, loss of E-cadherin expression was the only significant independent prognostic factor that was able to predict recurrence in patients with low-grade non-invasive UTUC, requiring hence an accurate follow-up [8]. Interestingly, in Velickovic et al. study [14], the loss of E-cadherin expression was correlated with the advanced stage of sporadic urothelial carcinomas as well as in patients with endemic nephropathy. However, Reis et al. [13] contradicted all previous studies and considered that the overexpression of E-cadherin is related to tumor recurrence and disease-free survival rates.

There were only a few studies of HER2 expression in UTUC [15, 16, 29, 30]. In the present study, we did not detect any HER2 positivity in all investigated cases as described in the early study of Bjerkehagen et al. [29], while only a low rate of HER2 overexpression and *HER2* amplification was reported by Langner et al. [16], suggesting that HER2-targeted therapy would be beneficial for only a small number of patients. Likewise, as *HER2* amplification and overexpression were rare events and more common in patients with high-grade tumors, lymph node invasion, and an inverted growth pattern, Vershasselt-Crinquette et al. [15] and Ehsani et al. [30] considered that only those patients may be potential candidates for Trastuzumab therapy.

## Conclusion

To the best of our knowledge, our study constitutes the first survey investigating p53, p27, Ki-67, E-cadherin, and HER2 expression and its clinicopathological significance among UTUC patients from Africa and the Arab world. Based on our results, the significant association of the positive surgical margin and the p53 expression with the RFS of patients clearly suggests their prognostic significance in UTUC. For the remaining clinicopathological features and the expression of p27, Ki-67, E-cadherin, and HER2, no significant association with patient RFS was identified. More advanced multicenter studies, using much larger series, are required to further explore these rare and aggressive tumors.

## Data Availability

Not applicable.

## Abbreviations

HER2: Human epidermal growth factor receptor type 2
pT: Pathology tumor
RFS: Recurrence-free survival
UTUC: Upper urinary tract urothelial carcinomas
WHO: World Health Organization
